# Supplementary material for: The Impact of Nutrition-Based Interventions on Nutritional Status and Metabolic Health in Small Island Developing States: A Systematic Review and Narrative Synthesis
Source: Nutrients. 2022 Aug 26;14(17):3529. doi: 10.3390/nu14173529 (PMC9460279; doi:10.3390/nu14173529)
Supplement: Supplementary file 1 [file nutrients-14-03529-s001.zip › nutrients-1847562-supplementary.pdf]

Supplementary Material

Figure S1. Risk of bias (RoB) overview for individual studies (*n*=50).

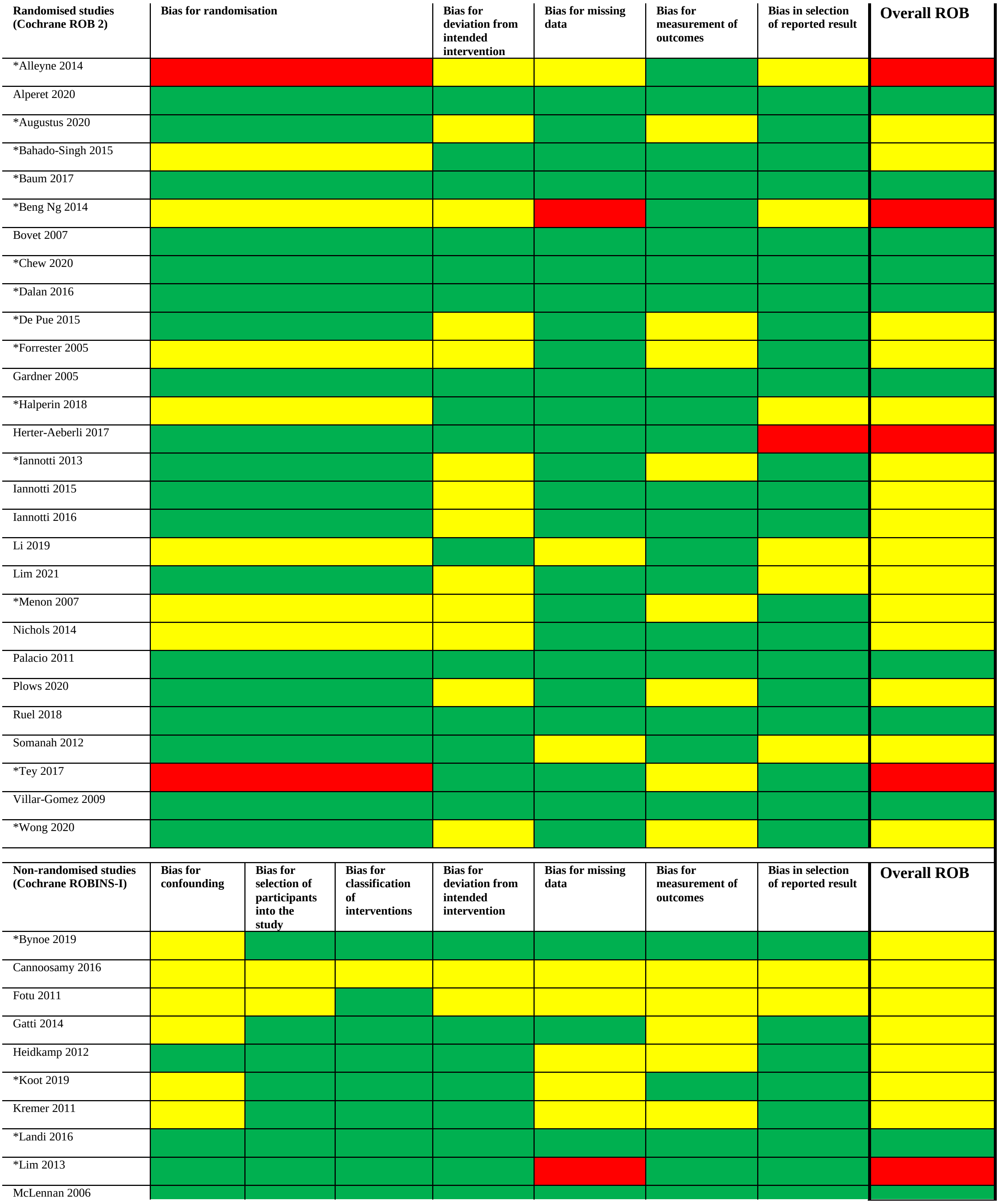

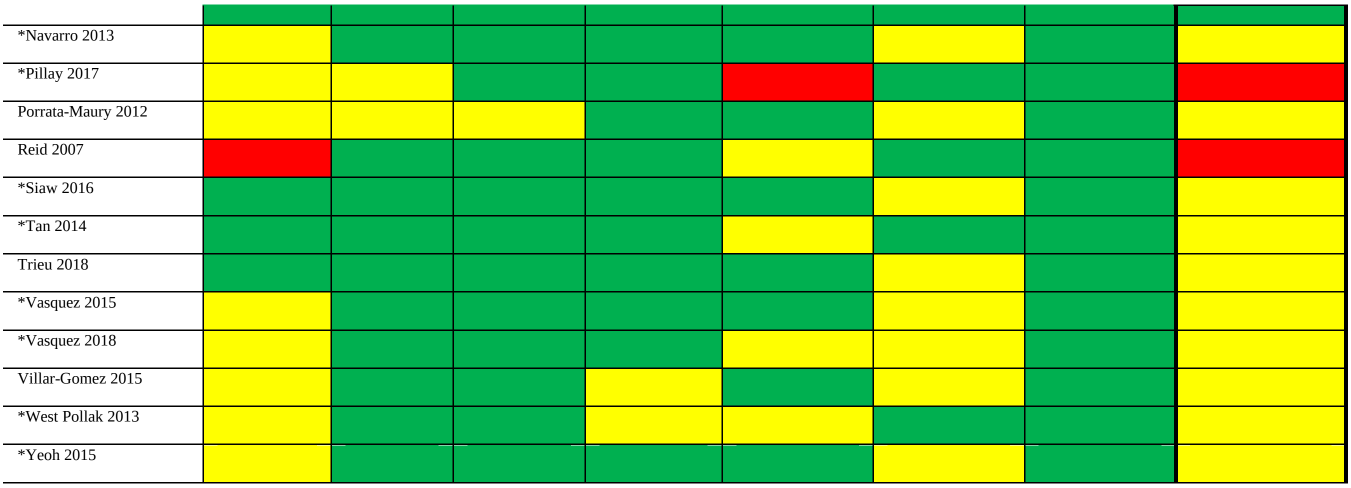

Key: green = low risk, yellow = moderate/some concern risk, red = high/serious risk
*Indicates the effective studies

Table S2: Overview of multifaceted interventions by level, setting and intensity (*n*=21)

Ref	Region	Intervention Description	Intervention Level	Setting	Participant Intensity
57	Pacific	Impact of intervention to support diabetic self-care	Household	Clinic	Low
59	Pacific	To build the capacity of communities and schools to create their own solutions for promoting healthy eating, physical activity, and healthy weight gain	Community	School	High
61	Pacific	To prevent rises in obesity by modifying school food.	Individual	School	High
62	Caribbean	To assess impact of multimodality weight gain prevention intervention	Individual	School	High
63	Caribbean	24-wk intervention included a lipid-based nutrient supplement and education	Individual	Clinic	High
68	AIMS	To assess the potential effectiveness and feasibility of GlycoLeap	Individual	Clinic	High
69	Pacific	Community-based intervention to promote healthy eating and regular exercise; reduce overweight	Community	Community	Unclear
70	Pacific	Impact of multifaceted intervention on malnutrition.	Individual	Clinic	High
73	AIMS	To restore optimal weight in postnatal women with a recent history of GDM.	Individual	Clinic	High
77	Caribbean	The effectiveness of a school-based intervention on dietary knowledge.	Individual	School	Low
79	Pacific	Intervention aimed at reducing population level salt intake.	National/Policy	General Population	High
83	Caribbean	Impact on child growth. 9 to 18 months food-based intervention.	Household	Community	High
86	AIMS	Impact of non-surgical weight loss program	Individual	Clinic	Low
88	Pacific	To assess the effect of the salt reduction strategy on population.	National/ Policy	General Population	Unclear

89	AIMS	To assess the effectiveness of a national corporate team-based weight loss intervention.	Individual	Workplace	Medium
90	AIMS	To promote individuals to lose weight in a healthy way through a multi-component	Individual	Workplace	NA
91	Caribbean	To evaluate whether the addition of Viusid to a hypocaloric diet and exercise would be associated with a greater histological improvement.	Individual	Clinic	Low
92	Caribbean	To evaluate the impact of a program of lifestyle changes through a hypocaloric diet.	Individual	Clinic	High
93	Caribbean	Lifestyle management of prediabetes and diabetes	Community	General Population	High
94	AIMS	To improve the health behaviours and outcomes among women aged 50 years and older	Individual	Community	High
95	AIMS	To study the changes in body composition and metabolic profile in Muslim patients	Individual	Clinic	Low

Region: Caribbean, Pacific, AIMS

Box 1: Search terms piloted in Medline

Search identification number	Search terms	Results
1	(Anguilla.tw) OR (Antigua.tw) OR (Antilles.tw) OR (Aruba.tw) OR (Bahamas.tw) OR (Barbuda.tw) OR (Barbados.tw) OR (Belize.tw) OR (Bermuda.tw) OR (Caicos.tw) OR (Caledonia.tw) OR (Caribbean.tw) OR (Cayman.tw) OR (Comoros.tw) OR ("Cook Islands".tw) OR (Cuba.tw) OR (Curacao.tw) OR (Dominica.tw) OR (Dominican.tw) OR (Fiji.tw) OR (Grenada.tw) OR (Grenadines.tw) OR (Guadeloupe.tw) OR (Guam.tw) OR (Haiti.tw) OR (Jamaica.tw) OR (Kiribati.tw) OR (Lesotho.tw) OR ("Saint Lucia".tw) OR ("St. Lucia".tw) OR (Maarten.tw) OR (Madagascar.tw) OR (Maldives.tw) OR (Marshall.tw) OR (Martinique.tw) OR (Mauritius.tw) OR (Melanesia.tw) OR (Micronesia.tw) OR (Montserrat.tw) OR (Nauru.tw) OR (Nevis.tw) OR (Niue.tw) OR (Pacific.tw) OR (Palau.tw) OR (Papua.tw) OR (Polynesia.tw) OR (Principe .tw) OR (Kitts.tw) OR (Samoa.tw) OR ("Sao Tomé".tw) OR (Seychelles.tw) OR (Singapore.tw) OR ("small island developing states" .tw) OR (Solomon.tw) OR (Suriname.tw) OR (Timor-Leste.tw) OR (Tonga.tw) OR (Trinidad.tw) OR (Tobago.tw) OR (Tokelau.tw) OR (Turks.tw) OR (Tuvalu.tw) OR ("Puerto Rico".tw) OR (Marianas.tw) OR (Martinique.tw) OR (Vanuatu.tw) OR (Verde.tw) OR ("Saint Vincent".tw) OR ("St. Vincent".tw) OR ("Virgin Islands".tw)	113648
2 (These terms are based on keywords from WHO Global Health Observatory Data repository and	deficien*.tw OR hydration.tw OR malnutrition.tw OR anemia.tw OR anaemia.tw OR stunt*.tw OR wasting.tw OR folate.tw OR haemoglobin.tw OR calcium.tw OR iron.tw OR vitamin.tw OR mineral.tw OR zinc.tw OR ("hepatic enzymes".tw) OR creatinine.tw OR urea.tw OR ("uric acid".tw) OR ("C-reactive protein".tw) OR ("inorganic	3,540,607

other systematic reviews [de Carvalho, 2015; Novotny,2015]	sulphate".tw) OR ketone*.tw OR potassium.tw OR sodium.tw OR albumin.tw OR phenols.tw OR nutrition*.tw OR nutrient*.tw OR diet.tw OR obes*.tw OR BMI.tw OR ("body mass index".tw) OR weight.tw OR diabetes.tw OR insulin.tw OR glucose*.tw OR ketosis.tw OR cholesterol*.tw OR lipid*.tw OR triglyceride*.tw OR lipoprotein*.tw OR hypertension.tw OR systolic.tw OR diastolic.tw OR hyperlipidaemia.tw OR hyperlipidemia.tw OR cardiovascular.tw OR ("heart disease".tw) OR ("waist circumference".tw) OR (" hip circumference".tw) OR ("waist to hip ratio".tw) OR ("waist to height ratio".tw) OR ("body fat".tw) OR ("visceral fat".tw) OR ("muscle mass".tw) OR adipos*.tw OR ("metabolic syndrome".tw)	
3	assessment.tw OR intervention*.tw OR evaluation.tw OR experiment*.tw OR program*.tw OR strateg*.tw OR initiative.tw OR polic*.tw OR project.tw OR plan.tw OR method*.tw OR treatment.tw OR guideline.tw OR tax*.tw OR levy.tw OR incentiv*.tw OR price*.tw	13,109,370
4	1 and 2 and 3	94,178
5	2000:2020. (timeframe)	67,035

Table S1: Study characteristics for all included studies (*n*=50).

Study ref	Study authors	Study design	Region/Country	Population/ subgroup studied	Intervention delivery	Sample size (loss to follow up)	Intervention	Control	Measured outcomes*	Tools	Effectiveness on outcomes of interest (to this review)
Multifaceted interventions											
57	DePue 2013	Cluster-randomized parallel group trial	Pacific / American Samoa	18 years and older with Type 2 Diabetes - clinic	Nurse community manager and community health workers	IG:104 (9%) CG:164 (10%)	Education – nutrition, physical activity, stress management, self-medication management and monitoring health	Nothing (included in a wait list to join the intervention after a year)	Metabolic- **HbA1c**	DCA 2000+ analyzer	**HbA1C** – At 12 months, mean HbA1c was statistically significantly lower among participants ($p<0.01$), compared with usual care, after adjusting for confounders
59	Fotu 2011	Non-randomized controlled before/after study	Pacific / Tonga	Secondary school children 11-19 years	Research assistants	IG:2610 (25%) CG:2182 (36%)	Nutrition activities: school canteen policies, plant and maintain community garden, healthy cooking classes. Physical activity component.	Nothing	Metabolic - **Weight change**	2007 WHO Reference standards for age/gender specific body mass index (BMI) centiles and cut-offs	**Weight change** – No significant changes, contrarily both intervention and comparison groups showed similar large increases in overweight and obesity prevalence (10.1% points, *n* = 815; 12.6% points, *n* = 897 respectively)
61	Gatti 2015	Non-randomized controlled before/after study	Pacific/ French Polynesia	Students aged 10 to 18	Nutrition: Canteen Staff Physical Activity: Mataura College physical education teachers and volunteers of the Island's Va'a club	IG:240 CG:90 (7%)	Balanced diet based almost exclusively on local agricultural products and fishing by the island community and documentation on healthy lifestyles	Nothing	Metabolic - **Weight change**	Freestanding stadiometer and bioelectrical impedance analyzer.	**Weight change** - Compared with the controls, the adjusted difference in weight change with intervention was 3.4 kg (95% CI, 4.3 to 2.5) and was the highest for comparison with the residents (4.9 kg; 95% CI, 6.1 to 3.8). This was a statistically significant difference in weight change ($p<0.001$).
62	Halperin 2019	Individually randomized parallel-group trial	Caribbean / Puerto Rico	University students aged 18 and 19	Health behaviourist, PhD expert in nutritional interventions, and an experienced yoga and meditation instructor.	IG:19 CG:21 (2.5%)	10 weekly peer support groups: identified and promoted dietary and other lifestyle changes, including physical activity and techniques to reduce stress and increase	Basic standard of care for students expressing concerns about managing their weight: i.e., provision of basic educational resource materials	Metabolic - **BMI**	Weight -a digital scale (ZB21, Ozer, SanDiego, CA) and height - a portable stadiometer (HM201M, Charder, Taichung City, Taiwan).	**BMI** - Significant reduction in BMI among the experimental participants compared with the control group at 10 weeks. Likelihood ratio test ($x2 = 26.9$; degrees of freedom [DF] =2; ($p < 0.001$), within each arm separately, BMIs differed after 6 months (experimental: $x2 = 15.9$, DF = 2, ($p < 0.001$);

							mindfulness.	on diet and physical activity			control: x2 = 10.7, DF = 2, (p = 0.004).
63	Heidkamp 2012	Non-randomized controlled before/after study	Caribbean / Haiti	Infants 6 to 12 months exposed to HIV and mothers - clinic	Two nutrition counsellors and two study assistants	IG:82 (13%) CG:294	A lipid-based nutrient supplement, education, promotion of existing clinical services, and social support (24 weeks)	Nothing (control was based on medical records)	Nutrition - **Weight change for age / height z scores**	WHO 2006 Growth Standard. Length was measured using an Easy-Glide Bearing Infantometer, Perspective Enterprises) and weight was measured using a digital scale (Tanita Model 1583)	**Weight change for age / height z scores** - The intervention and historical control groups did not differ significantly at age 6 months (wk 0) or 12 months (wk 24), the intervention group had a lower prevalence of underweight and stunting than the historical control group (weight-for-age Z-score,22 SD: 6.8 vs. 20.8%, p = 0.007; length-for-age Z-score, 22 SD: 9.6 vs. 21.2%, p = 0.029).
68	Koot 2019	Non-randomized Uncontrolled before and after study (pre/post test study)	AIMS / Singapore	Adults with diabetes aged 21 to 70	Research coordinator	100 (17%)	24 weeks of access to GlycoLeap, a glucometer kit, a wireless weighing scale, a resistance band for strength training; printed instruction guides (Glyco app and devices) and healthy lifestyle guidebooks	No control group	Metabolic - **HbA1c**	Accu-Chek Performa (F. Hoffmann-La Roche Ltd) glucometer kit with lancets and test strips	**HbA1c** - participants' HbA1c levels were 1.3 percentage points lower at follow-up compared to baseline (7.6% [95% CI 7.2-7.9] vs 8.9% [95% CI 8.5-9.2], p<0.001) and 49 of 83 participants (59%) achieved a ≥1 percentage point reduction in HbA1c levels.
69	Kremer 2011	Non-randomized controlled before/after study	Pacific / Fiji	Secondary school students in forms 3-6 aged 13 to 18	Unclear	IG:3530 (67%) CG:6255 (55%)	Children encouraged to engage in healthy lifestyle activities: reduce television viewing, reduce energy-dense snacks and drinks and increase fruit and water intake, active play, and walking. Parents and schools encouraged to support.	Nothing	Metabolic – **BMI for age**	Unclear	**BMI for age -** At follow up the intervention resulted in no difference in BMI across groups (p>0.05).

70	Landi 2017	Non-randomized Uncontrolled before and after study (pre/post test study)	Pacific / Papua New Guinea	Inpatient infants 14 to 36 months	Parents with the guidance and collaboration of hospital staff (nurses)	125 (5.6%)	Nutrition management seminars and at least 130 ml/kg/day, which (about 69 kcal/kg/day) of a standard full-cream milk formula or 100 kcal/kg/day of a fortified milk-oil formula (over 2 months)	No control group	Nutrition - **Weight change for age / height z scores**	Data were collected directly from patients' medical records and monitoring charts, and by direct measurement and weighing of the patient	**Weight change for age / height z scores** - Weight gain increased to 5.56 g/ kg/day (IQR -3.7-12.0) and 10.19 g/kg/day (IQR 0-16.0) in the first and second post-intervention follow-up. This represented a significant increase in the average weight gain between the baseline and follow-up surveys (Kruskal-Wallis test p=0.013).
73	Lim 2021	Individually randomized parallel-group trial	AIMS/ Singapore	Postnatal women with history of GDM (≥21 years)	Study team consisting of dietitians, physiotherapists, and occupational therapists.	IG: 101 CG: 99 (9%)	App called Nutritionist Buddy (nBuddy), face to face support in how to use the app, and information booklet. App has information on healthy eating, exercise, and breastfeeding.	Standard care - follow up clinician appointment at 6 weeks postpartum, routine check, dietary advice and repeat OGTT performed.	Metabolic - **Weight Change**	Not stated	**Weight change** - No statistically significant differences in weight reduction at four months (although intervention arm achieved a mean difference in weight reduction of 1.05 kg compared with the control arm (95% CI 0.14 to 2.24; p=0.08).
77	Nichols 2014	Individually randomized parallel-group trial	Caribbean / Trinidad and Tobago	6th year primary school pupils	Trained staff	IG: 299 CG:280 (20%)	A specifically designed curriculum consisting of lessons on nutrition and physical activity based on Bloom's mastery learning model.	Nothing	Metabolic - **BMI**	Electronic weighing scale (model 770; Seca Corp., Hanover, MD, USA) and height: portable stadiometer (Seca Corp.) CDC age and gender-specific cut-off values were used to categorize BMI risk percentiles.	**BMI** - There was no significant decrease in BMI (p>0.05).
79	Pillay 2017	Non-randomized Uncontrolled before and after study (pre/post test study)	Pacific / Fiji	Adults 25-64 from the general population	Health workers, media, community leaders.	272 (22.4%)	Consumer awareness campaign on salt and health including nutrition education materials	No control group	Metabolic - **Blood pressure**	WHO STEPS surveillance protocol	**Blood pressure** - Significant difference in diastolic blood pressure from baseline to follow up (p=0.037).

83	Ruel 2008	Cluster-randomized parallel group trial	Caribbean / Haiti	Infants and toddlers 6 to 59 months - community	Health care and community workers	IG:788 CG:792 (6.3%)	Monthly food ration: 8 kg of micronutrient-fortified wheat soy blend and 2 kg of oil, and ration for general household consumption of 10 kg of wheat-soy blend and 2·5 kg of lentils (6 to 23 months).	Same food rations as the intervention group (6 to 59 months)	Nutrition - **Weight change for age / height z scores**	Not specifically stated, presumably stadiometers and weight scales	**Weight change for age / height z scores** - At follow-up, stunting, underweight, and wasting (using WHO 2006 reference data) were 4-6 percentage points lower in preventive than in recuperative communities; and mean anthropometric indicators were higher by +0·14 Z scores (height for age; p=0·07), and +0·24 Z scores (weight for age and weight for height; p<0·0001).
86	Tan 2014	Non-randomized Uncontrolled before and after study (pre/post test study)	AIMS / Singapore	Adults aged 18-65 years - clinic	Physicians and a team of dieticians, physiotherapists and occupational therapists	58 (33%)	Five consultations with a physician, five consultations with a dietician, two supervised gym sessions with physiotherapists and two group sessions with occupational therapists	No control group	Metabolic - **Weight change**	Not specifically stated	**Weight change** - Achieved a mean weight loss of 2.1 kg. Less than 15% of patients could be considered to have achieved successful outcomes in terms of weight loss (i.e. weight loss of ≥ 5%). Two patients managed to achieve a weight loss of ≥ 10% upon completion of the programme.
88	Trieu 2018	Non-randomized Interrupted time series studies and repeated measures study	Pacific / Samoa	Adults 18-64 from the general population	Government (mainly Ministry of Health)	780 (49%)	Changes to Food Act, food industry regulations on salt targets, education interventions, and increased awareness through campaigns and resources.	No control group	Metabolic - **Weight change**	Digital Omron scales and Seca portable stadiometer. Automatic BP monitor with an appropriately sized cuff. One 24-h urine sample - no further detail.	**Weight change** - No significant changes to anthropometric measures.
89	Vasquez 2015	Non-randomized Uncontrolled before and after study (pre/post test study)	AIMS/ Singapore	Adults 18 years and older - work place	Dieticians, fitness or trainers, accessors.	785 (10%)	21 physical activity sessions, 3 nutrition education sessions, and 3 fitness assessments over a period of 12 weeks.	No control group	Metabolic - **BMI**	Tanita digital weighing scale. At the end of the intervention, a BMI Machine (Avamech B1000-M) was also used.	**BMI** - The average mean change in BMI was -1.32 kg/m^2 (-1.51, 1.13) at the 12 weeks post baseline, and -1.50 kg/m^2 at the end of the intervention. Average percentage change in BMI was -4.37%.

90	Vasquez 2018	Non-randomized controlled before/after study	AIMS / Singapore	Adults 18 to 69 years and older - work place and communit y	Not clear. 'Motivators' who were participants from previous rounds delivered support in latter rounds.	IG: 2951 CG: 677 (37%)	Informative and interactive sessions on physical activity and nutrition education, supported by behavioral modification strategies.	Wait list – to enrol in intervention	Metabolic - **BMI**	2009 - Tanita digital weighing scale for baseline and BMI Machine (Avamech B1000-M) - post. 2010 the BMI Machine (Avamech B1000-M) – both. 2012 and 2013 standardised digital weighing scales (Omron HN286) – both.	**BMI** - Within intervention groups participants showed significant reductions in BMI. No analyses were conducted to explore whether there were significant differences between arms.2009 mean reduction in BMI. Workplace: -1.5 (-1.69, -1.30). 2010 mean reduction in BMI. Workplace: -1.44 (-1.65, -1.22); Community: -1.35 (-1.48, -1.22). 2012 mean reduction in BMI. Community: -0.93 (-1.06, -0.80). 2013 mean reduction in BMI. Community: -1.00 (-1.10, -0.90).
91	Villar-Gomez 2009	Individually randomized parallel-group trial	Caribbean / Cuba	Adults 18 to 70 years - clinic	Not clear, but implies 'physicians and study coordinators'	IG: 30 (6.7%) CG: 30 (10%)	Three oral sachets daily of viusid, plus hypocaloric diet and exercise<br>Diet based on by calories: 64% CHO, 22% (<10% saturated); 14% protein. Exercise: 40mins walking or jogging 5 days per week	Diet and exercise only as described for th eintervention group.	Metabolic - **BMI**	Not stated	**BMI** - In both IG and CG there were improvements in BMI. In the IG, difference in BMI at 6 months was (p>0.001) and in the CG difference in BMI at 6 months was (p>0.01).
92	Villar-Gomez 2015	Non-randomized Uncontrolled before and after study (pre/post test study)	Caribbean / Cuba	Adults 18 years and older – clinic	Medical staff	293 (8.2%)	Recommendation for a low-fat hypocaloric diet that was 750 kcal/d less than their daily energy need, distributed in carbohydrates 64%; fat 22%, with <10% of saturated fatty acids of total daily calories; and protein 14%	No control group	Metabolic - **Weight change**	Body weight (kg) was measured by calibrated hospital scales	**Weight change** - All subjects who completed the study had a mean weight loss of 4.6 ± 3.2 kg, which corresponds to a reduction in daily energy intake of approximately 413 ±133 kcal .

93	West Pollak 2014	Non-randomized Uncontrolled before and after study (pre/post test study)	Caribbean / Dominican Republic	Adults (age range not stated) communit y / general population	Community leaders	76 (22.4%)	Quarterly physician visits, and access to a nurse practitioner, an exercise physiologist, a nutritionist and a diabetes educator.	No control group	Metabolic - **HbA1c**	A1CNow+ point-of-care test (Bayer HealthCare, Sunnyvale, CA, USA)	**HbA1c** - At 6 months, there was an average decrease of 0.49% in HbA1c.Mean HbA1c was 7.28 (6.67, 7.89) p=0.015. At 6 months mean HbA1c was 7.06 (6.46,7.66) p-0.005. It was similar between patients who were on medical therapy at the start of the study and those who were not.
94	Wong 2021	Cluster-randomized parallel group trial	AIMS / Singapore	Women - 50 years and older (communi ty)	Recreation centre managers and program ambassadors	IG: 351<br>CG: 331<br>14.96%	Educational and skill sessions, along with telephone support. Distributed and explained printed resources (health calendar, PA and nutrition booklets, and recipe booklet)	The control group participants only received a falls prevention booklet.	Metabolic - **Blood Pressure**	Systolic and diastolic BP were measured 3 times using an Omron electronic sphygmomanomet er on the participant's upper arm at 1-minute intervals.	**Blood pressure** - The diastolic and systolic BP in the intervention participants were found to be significantly improved postintervention with decreases of 3.54 and 3.68 mm Hg, respectively (0 < 0.001).
95	Yeoh 2015	Non-randomized Uncontrolled before and after study (pre/post test study)	AIMS / Singapore	Muslim patients with diabetes mellites - 18 years and over	No information	29 (13.8%)	All subjects underwent educational sessions with a physician and a dietician according to the recommendations of the American Diabetes Association on management of diabetes during Ramadan.	No control group	Metabolic – **HbA1c**	Not specified	**HbA1c** - At the end of Ramadan, HbA1c improved signifi cantly (8.6 ± 2.4 to 8 ± 2.3%, P = 0.017).
Supplements / fortified foods											
50	Baum 2017	Cluster-randomized parallel group trial	Caribbean / Haiti	Infants / children ages 6-59 months - communit y	Centre chiefs and nurses	IG: 262<br>CG: 259<br>(no details)	Powders distributed to households enrolled at credit centres for children to take. Education seminars for parents that covered nutrition, extended	Micronutrient powders after three-month follow-up at the conclusion of the study.	Nutrition - **Haemoglobin concentration**	Portable battery-run device (HemoCue Hb 201 + Analyzers) operated by trained nurses.	**Haemoglobin concentration -** The mean difference in hemoglobin concentration between children in the intervention group and those in the control group was 0.28 grams per deciliter (g/dL)-with a subsample of younger

							breastfeeding, detection of fever and risks of malaria.				children (under two years of age) showing greater relative improvement (0.46 g/dL). (p>0.05).
55	Chew 2021	Individually randomized parallel-group trial	AIMS / Singapore	Elderly 65 years and above	A healthcare services provider along with trained dietitians	IG: 406 CG: 405 (4.3%)	Two servings/day of ONS containing hydroxy-beta-methylbutyrate (HMB) with dietary counseling for 180 days . The supplement contained 262 kcal, 10.5 g protein, 8.5 g fat, 34.2 g carbs, 7.75 mcg (310 IU) Vitamin D3, and 0.74 g calcium (HMB) per serving.	Two servings/day of placebo supplement with dietary counseling. . Placebo supplement contained 60 kcal, 1.07 g protein, 1.21 g fat and 11.9 g carbs per serving	Nutrition - **Vitamin D Deficiency**	Immunochemistry analyzer COBAS e801 was used to determine 25-hydroxyvitamin D levels, and cut-off values based on Holick were used to ascertain vitamin D status	**Vitamin D Deficiency** - For the IG, the percentage of participants with vitamin D deficiency reduced from 20.0% at baseline to 6.3% at day 90 and 3.9% at day 180.Placebo group : unchanged. The odds of having better vitamin D status in the IG was significantly higher than the placebo group during the study (OR ¼ 4.23; 95% CI: 3.04, 5.90; p < 0.001). IG had significantly higher 25-hydroxyvitamin D levels than the placebo group at day 90 (32.12 ± 0.43 mg/L vs. 28.31 ± 0.43 mg/L; P < 0.001) and day 180 (31.86 ± 0.42 mg/L vs. 27.30 ± 0.43 mg/L; p < 0.001)
56	Dalan 2016	Individually randomized parallel-group trial	AIMS / Singapore	Adults 44 to 63 years – clinic	Study team (reviewed by an independent clinician)	IG: 33 CG: 31 (4.7%)	Vitamin D pills (4000 IU (n=21) ; 2000 IU (n=12))	Placebo pills (4 pills (n=18); 2 pills (n=13)	Metabolic - **Endothelial function**	No information.	**Endothelial function** - At the final 16-week visit, the mean 25(OH)D was 31.6±9.5ng/mL (change of 14.3ng/mL; p<0.0001), mean adjusted calcium was 2.41±0.10mmol/L and iPTH was 4.5±2.1mmol/L At 16weeks, 23 out of 33 (70%; 95% CI: 53%-83%) patients achieved 25(OH) D>30 ng/mL (75 nmol/L).
60	Gardner 2005	Individually randomized parallel-group trial	Caribbean / Jamaica	Infants / toddlers 9 to 30 months – clinic	Community health workers	IG: 84 CG: 42 (10.5%)	Zinc supplementation consisted of 10 mg elemental zinc (the recommended dietary allowance for children aged 12 mo) as sulfate in a flavored syrup	Placebo (syrup only)	Nutrition - **Weight for age / height z scores**	Weights and lengths or heights were measured by using standard techniques by 2 research assistants.	**Weight for age / height z scores -** Supplementation had no significant effect on any of the anthropometric indexes.

64	Herter-Aeberli 2017	Individually-randomized cross over trial	Caribbean / Haiti	Mothers between 18 and 45 years and children between 2·5 and 5 years - community	Study staff	44 (4.5%)	Women - two bread rolls (approx. 94 g) with 30 g of cream cheese and 410 g of Sékola Citron. Children - one bread roll (approx. 47 g) with 15 g of cream cheese and 160 g beverage above. FeFum (57Fe) was added as a powder and NaFeEDTA was added as a liquid to bread.	The participants served as their own controls and all received Test Meal A (FeFum)	Nutrition - **Iron absorption**	Total circulating Fe was calculated based on blood volume, which was estimated based on weight and height using the equation by Brown et al.	**Iron absorption** - Fractional absorption was not significantly different comparing mothers with children for either test meal. For both groups, fractional absorption was significantly higher from meal B (NaFeEDTA) compared with meal A (FeFum) (P=0·029). Absorption from test meal C (FeFum + NaFeEDTA) was not significantly different from test meals A or B (P>0·05), and showed an intermediate value.
65	Iannotti 2014	Individually randomized parallel-group trial	Caribbean / Haiti	Infant-mother pairs. Infants less than 1, sample aged 6-11 months	Nurses	IG1:196; IG2:202 CG:191<br><br>At visit 6 - 25%; Visit 7 - 29%	Two groups:<br>1) a 3-mo lipid-based nutrient supplement (LNS); or<br>2) a 6-mo LNS. The LNS provided 108 kcal ; vitamin A, vitamin B-12, iron, and zinc at 80% of the recommended amounts. Mother-child pairs in LNS trial arms received an exact monthly supply of LNS at their clinic visits	All 3 groups including control received the MSPP standard of care of the IP of well-baby services. They also received a full 6-mo supply of the LNS.	Nutrition - **Stunting**	Weights - Seca Model 874 Electronic Digital Scale Infant/Child ShorrBoard (Shorr Productions LLC) – heights. Mothers - ShorrBoard Portable Height-Length Board (Shorr Productions LLC).	**Stunting** - LNS supplementation for 6 mo significantly increased the length-for-age z score (6SE) by 0.13 6 0.05 and the weight-for-age z score by 0.12 6 0.02 compared with in the control group after adjustment for child age (p= 0.001).
66	Iannotti 2015	Cluster-randomized parallel group trial	Caribbean / Haiti	School children 3 to 13 years old	Enumerators	1167 (4.4%)	Mamba once per day for a total of 100 days.	Tablet Yo schools once per day for a total of 100 days. Received Mamba next academic year.	Nutrition - **Weight for age / height z scores**	The Seca Model 874 (Digital) 440-lbs 3 0.1-lb resolution scale and the ShorrBoard height measuring board were used to collect parent and child weight.	**Weight for age / height z scores** - The school children showed negative mean z scores throughout the study period across all groups. Univariate statistical differences in weight-for-age z score change from baseline to endline were evident for Mamba compared to Tablet

											Yo and Tablet Yo compared to control ($p < 0.05$).
67	Iannotti 2016	Cluster-randomized parallel group trial	Caribbean / Haiti	School-aged children 3 to 16 years	Teachers	IG: 170<br>CG: 151 (2.8%)	Vita Mamba once per school day from November 2014 to June 2015, for approx.. 26 weeks. Also received albendazole at baseline in November 2014	Albendazole at baseline in November 2014	Nutrition - **Anaemia**	Hb - Hemocue system and reported in grams per 100 milliliters (g/dL). Compared to World Health Organization (WHO) anemia cut-offs for anemia.	**Anaemia** - No statistical differences in change in HAZ, WAZ, and BMIz by treatment group were evident. Children ages 11 to 16 years showed the greatest reduction in HAZ score from baseline to endline compared to other age groups (ANOVA, $p = 0.01$). Hb concentration increased across all age groups in the Vita Mamba group and decreased by endline for all age groups of the control groups, reaching statistical significance in the children ages 11 to 16 years.
74	Mc Lennan 2006	Non-randomized Uncontrolled before and after study (pre/post-test study)	Caribbean / Dominican Republic	Children (mean age 29.6months) - clinic	The day hospital staff	88 (46%)	Vitamin A and folic acid supplementation, as well as anti-parasitic medication, were routinely administered. Rehydration strategies were employed as needed. Formula feeding WHO guidelines.	No control group.	Nutrition - **Weight for age / height z scores**	Weight for height Z-scores using the 2000 CDC growth data and the associated formula: : Z ¼ ½ðX=MÞ<br>L 1=LS	**Weight for age / height z scores** - Overall mean growth rate during rehabilitation up to 4 weeks after admission falls in the poor range. Slightly higher rates of growth were noted on attendance days vs. non-attendance days, though the difference was not statistically significant. 27% of the children achieved an overall growth rate of over 5 g/kg/day.
75	Menon 2007	Cluster-randomized parallel group trial	Caribbean / Haiti	Infants - 9 to 24 months - community	Health workers	IG: 254<br>CG: 161 (4%)	A 2-mo supply of Sprinkles for the infants / toddlers and a BCC intervention (for the mothers), which conveyed information on appropriate use of the Sprinkles.	WSB only, no sprinkles	Nutrition - **Anaemia**	Hemocue Hb point of care machine	**Anemia** - At first follow up (2 months) prevalence of anaemia fell in IG (52.3% to 28.3%) but not in CG (36.6% to 44.8%). At second follow up (9 months), prevalence 15.5% in int group - NB CG was given sprinkles after 2 months. Mean Hb increased in IG by 5.5 and 10.9 g/l at 1st and 2nd follow up. In

							Fortified WSB (wheat soy blend) for 2 consecutive months as well.				cont mean Hb fell by 1.0g/l at first follow up.
85	Somanah 2012	Individually randomized parallel-group trial	AIMS / Mauritius	Adults 26-60 – general population	Unclear	127 (20%)	1 standard sachet of FPP® dissolved in half a glass of warm water twice daily before meals for 14 weeks followed by a 2 week wash out period of consuming the same amount of water/day	Equivalent amount of water	Metabolic - **HbA1C**	Samples were analyzed using a fully automated clinical chemistry analyzer (Olympus AU480, Beckman Coulter® Inc.)	**HbA1C** - No change in Fasting blood glucose and glycated hemoglobin (HbA1c)
Dietary Change											
48	Augustus 2021	Individually randomized parallel-group trial	Caribbean / Trinidad and Tobago	Adults 18 years and over – clinic	Trained nurses. Assistance if required from family or live-in helper.	IG: 20 CG:20 (10%)	A seven day cyclic altered KD offered over a 16-week period. Meals contained approximately 10% (50g) carbohydrate, 15% (75g) protein and 75% (167g) fat (mainly medium chain triglycerides, ~2000kcal.	No ketogenic diet - to consume their usual diet with minor alterations being made to ensure that their daily energy and nutrient requirements were met.	Metabolic - **BMI**	Scales and stadiometer - standard formula to calculate BMI	**BMI** - Statistically significant ($p<0.05$) mean change reduction (mc = 1.324). For between study differences overtime BMI showed significant differences ($p=0.042$).
49	Bahado-Singh 2015	Individually randomized parallel-group trial	Caribbean / Jamaica	Adults 25-65 – clinic	Participants (self administered) with dietary advice / meal plan to be followed	IG: 32 CG: 33 (18%)	Low to Intermediate Glycaemic Index Caribbean Diet	High Glycaemic Index Caribbean Diet	Metabolic – **HbA1C**	HbA1C was determined by boronate affinity and high-performance liquid chromatography (HPLC) using the Primus-PDQ Plus Analyzer	**HbA1C** - There was a greater decrease in A1C between the low-intermediate-GI group and the high-GI group (9.03% and 4.03% respectively; $p < 0.05$). This percentage decrease represents an absolute decrease in A1C units of 0.84% in the low-intermediate-GI group which was lower than that observed for the high GI group, which had a decrease of 0.35%.
53	Bynoe 2020	Non-randomized Uncontrolled	Caribbean / Barbados	Adults aged 20-69 years	Family practitioner	25 (4%)	8-week liquid (760 calorie) diet.	No control group	Metabolic- **Fasting**	Fasting glucose was measured on capillary blood	**Fasting Blood Glucose** - Six months after the end of the low-calorie diet phase, 9 of 24

		before and after study (pre/post test study)		(Type 2 diabetes)					**Blood Glucose**	using a Hemocue (Angelholm, Sweden) 201 RT analyser.	participants (37.5%) who were followed up had a FPG < 7.0 mmol/l. The decrease in mean HbA1c was statistically significantly (p>0.001) between baseline and week 8 [9 mmol/mol (95% CI 4, 15);0.9% (95% CI 0.3, 1.4)]. It increased between week 8 and the end of the follow-up such that the mean decrease at month 8 compared with baseline was 7 mmol/mol (95% CI 4, 18; p= 0.19) [0.6% (95% CI 0.3, 1.6]
58	Forrester 2005	Individually-randomized cross over trial	Caribbean / Jamaica	Men - 25 to 55 years old – general population	Research nurses from the Tropical Metabolism Research Unit (TMRU)	56 (0%)	A low-salt or high-salt diet for 3 weeks, followed by a 2-week washout period and a crossover phase for an additional 3 weeks.The low-salt diet was designed to achieve a 50 mEq reduction in sodium while the supplements were anticipated to result in a 50 mEq increase in baseline sodium	It was a cross-over trial	Metabolic - **Blood pressure**	Standard mercury manometer Omron automatic device	**Blood pressure** - The mean change in manual systolic and diastolic BPs between the low-salt and high-salt phase was 5.1 (2.3, 8.0) and 2.2 (-0.7, 5.1) mmHg , respectively after adjustment for covariates.
78	Palacios 2011	Individually randomized parallel-group trial	Caribbean / Puerto Rico	Obese adults 21-50 years old	The research team	IG: 20 CG: 10 (12%)	High dairy group - four daily servings of dairy products with a dairy calcium intake goal of 1200-1300 mg/d. High elemental calcium group - consume their usual diet plus a calcium supplement of 600 mg/d .	Consume their usual diet plus a placebo tablet, for a total calcium intake goal of <700 mg/d. Subjects were instructed to consume one tablet daily with lunch or dinner.	Metabolic - **BMI**	Body weight was measured monthly with a calibrated scale (Detecto Inc., Northbrook, IL, USA). Height was measured at baseline using a wall-mounted tape.	**BMI** - No significant group effects were observed on the mean 21-wk change in weight (P = 0.77), BMI (P = 0.71), total body lean mass (P = 0.85), total body fat mass (P = 0.71), total body percentage fat (P = 0.49), trunk body fat mass (P = 0.71), trunk body percentage fat (P = 0.44).

81	Porrata-Maury 2012	Non-randomized Uncontrolled before and after study (pre/post test study)	Caribbean / Cuba	Adults 20-80 – general population	Unclear	65 (12%)	Dietary intervention based on a Macrobiotic vegetarian Ma-Pi 2 diet for 21 days, with food service daily (breakfast, lunch, dinner, and snacks), prepared by expert Ma-Pi macrobiotic cooks.	No control group	Metabolic - **Fasting Blood Glucose**	Serum glucose and lipids were determined using an Automatic Analyzer ELIMAT (SEPPIM, France), commercial reagents kits from HELFADiagnostics, Cuba.	**Fasting Blood Glucose -** Decreased significantly in both periods, after 21 days ($p>0.001$) and after 3 months ($p>0.0001$).
84	Siaw 2016	Non-randomized Uncontrolled before and after study (pre/post test study)	AIMS / Singapore	Adults - 21 years and older (Type 2 diabetic patients)	Self delivered	Total 5,172. 397 - G1 2,877- G2 and 1,898 - G3 (0%)	All three groups fasted during Ramadan	No control group	Metabolic - **HbA1C**	The CDMS was used (venous blood). This is an electronic, enterprise-wide chronic disease database with patient information across the healthcare clusters of three hospitals and nine primary care clinics.	**HbA1C** - The mean HbA1c values of groups 1 and 2 decreased by 1.4% and 0.1%, respectively, between the pre-Ramadan and Ramadan periods $p < 0.001$. Not sustained in the post-Ramadan period. From Ramadan to post-Ramadan periods, there were an insignificant increase in group 1 (0.1%, $p > 0.05$) and a significant increase in group 2 (0.2%, $p < 0.001$). The mean HbA1c values for group 3 increased steadily by 0.2% ($p < 0.001$).
Nutrition Education											
51	Beng Ng 2014	Individually randomized parallel-group trial	AIMS / Singapore	Adults 21 to 75 years – clinic	Not clear. Presumably primary health care workers	IG: 112 CG: 88 (69%)	Diet: Written exercise prescription with eventual target of 60 min five days a week. Weight control: Educational materials.	Usual advice through primary care. General dietary advice.	Metabolic - **BMI**	Not clearly stated.	**BMI** - The mean BMI decline was greater in the intervention than control group [0.71 (SD=1.17) and 0.08 (SD=0.90) kg/m2 respectively, $p=0.021$], and remained statistically significant after adjusting for age, gender and baseline BMI ($p=0.041$).
54	Cannoosamy 2016	Non-randomized Uncontrolled	AIMS / Mauritius	Housewives 30	No information.	200 (0%)	A lecture with information on general nutrition;	No control group	Metabolic - **BMI**	Scales and stadiometers	**BMI** - The mean body mass index of the participants was 22.5 ± 3.87 (pre-test), 22.5 ±

		before and after study (pre/post-test study)		years and older			the food groups; portion/serving sizes; calories: meaning and importance; how to plan a healthy diet; and on fruits and vegetables, that is, the recommended intake, their importance, and benefits to health. Material also.				3.87 (post-test) and 22.4 ± 3.85 (follow-up). The change in body mass index after the nutrition intervention was not significant (p = 0.751) where 10.5% of housewives had a decreased body mass index after the nutrition intervention, 10% had a greater body mass index, and the majority, that is,79.5, had unchanged body mass index values.
71	Li 2019	Individually randomized parallel-group trial	AIMS/ Singapore	Adults 21 years and over - clinic	Dietitians	IG: 15 CG: 15 (14%)	Free food-coaching smartphone app for 8 weeks - upload food images and receive real-time and detailed food coaching comments and guidance from dietitians between 8am-8pm.	No intervention / app	Metabolic - **Weight Change**	No information	**Weight Change** - no significant mean difference between intervention and control group in weight gain at 8 week follow up (-0.08 (-1.80 to 1.63) [p = 0.92].
72	Lim 2013	Non-randomized Uncontrolled before and after study (pre/post-test study)	AIMS / Singapore	Adults 21 years and over - clinic	Dietitians	163 (0%)	An ambulatory nutrition support program which included phone calls on week 1, 2 and 4 month after discharge from hospital, dietetic outpatient appointments at 1 and 3 months post-discharge.	No control group	Metabolic - **Weight Change**	Subjective global assessment, the 7 points modfication of the 3 points SGA scale, digital Seca Scale, Harpenden skinfold caliper	**Weight Change** - Improvements in mean weight from baseline to five months post discharge (2.2 ± 4.7 kg, p>0.001)
76	Navarro 2013	Non-randomized controlled before/after study	Caribbean / Dominican Republic	Infants 13-24 months	Community health volunteers	IG: 226 (26%) CG: 337 (22%)	Group meetings with educational activity every fifteen days with other pregnant women, fortnightly and then monthly	Nothing	Nutrition - **Weight change for age / height z scores**	Weight mother/child electronic scales with a precision of 0.1 Kg (UNISCALE, UNICEF Supply Division)	**Weight change for age / height z scores** - Significant reductions of BMI-for-age Z-score (−0.31, 95%CI −0.49; -0.12' p=0.001) and of BMI-for-age > 85th percentile (0.43, 95%CI 0.23; 0.77, p=0.005) were observed

							home visits to support breastfeeding, vaccination, newborn care, danger signs, complementary feeding and other topics.			Length, Shorr length measuring boards accurate to 0.1 cm outcomes (Shorr Productions Growth Unlimited, Olney, Md., USA)	
80	Plows 2020	Cluster-randomized parallel group trial	Pacific / Kiribati	Students aged 18 years and older – school	Registered dietitian delivered presentation/education	IG: 37 CG: 26 (33%)	Installation of a water filter at the school. Each participant received a metal water bottle. A registered dietitian gave a 30-minute presentation on sugar reduction for the purposes of type 2 diabetes prevention	No intervention (until after the study was completed)	Metabolic - **Change in mean blood glucose**	Continuous glucose monitors	**Change in mean blood glucose** - No significant effect of intervention on any of the CGM measures. For mean glucose (mg/dL) at baseline and follow up: control 88.8(7.6);88.1(8.3) Intervention: 83.4 (8.90);85.72 (8.60) (p =0.40)
Specific Foods											
46	Alleyne 2014	Non-randomized controlled before/after study	Caribbean / Trinidad and Tobago	Adults 35 to 60 years	Not clear	45 (20%)	Cocoa-based drink or the placebo (in any order) three hours after BP monitoring	'Control' was the placebo, which was green water - using food dye.	Metabolic - **Blood Pressure**	Welch Allyn ambulatory BP monitor	**Blood Pressure** - Systolic BP fell by 'up to 18mmHg' (p=0.0001), and a smaller fall in diastolic BP. This was within an hour of taking the cocoa, and sustained for around 2.5 hours. BP changes in non-hypertensive cohort were 'small and not significant' (no data given)
47	Alperet 2020	Individually randomized parallel-group trial	AIMS / Singapore	Adults 35 to 69	Participants delivered the intervention with instructions from the study staff	IG: 62 CG: 64 (18.3%)	Four cups of coffee daily for 24 weeks. Each sachet of the coffee beverage, contained 26.3% of regular Nescafe mixed with 73.7% of a non-dairy creamer. Both the coffee and the	Placebo beverage, contained 32.5% of a colored nondairy creamer mixed with 67.5% of the same non-dairy creamer used for the coffee beverage. Both the coffee and the coffee-like beverage	Metabolic - **Insulin sensitivity**	Hyperinsulinemic-euglycemic clamp	**Insulin sensitivity** - Coffee consumption did not significantly change insulin sensitivity at 24 wk compared with placebo (Mbw = 4.0%; 95% CI: −8.3, 18.0%; p = 0.53)

							coffee-like beverage contained 30 kcal per sachet.	contained 30 kcal per sachet.			
52	Bovet 2007	Individually randomized cross over trial	AIMS / Seychelles	Adults 23 to 49 years – MoH volunteers	Not stated	25 (8%)	A fish oil enriched egg per day	A non-fish oil enriched egg per day	Metabolic - **TAGS**	TAGS level was measured with an enzymatic colorimetric GPO/PAP method (ABX Diagnostics)	**TAGS** - Triglyceride levels fell by around 16 to 18% when eating the fish oil enriched eggs compared to the normal eggs ($p<0.01$)
87	Tey 2017	Individually randomized cross over trial	AIMS / Singapore	Adults 21 to 35 years – general population	Two qualified researchers	19 (0%)	Participants consumed glucose (reference food) on three occasions and test fruits (guava bites, guava puree, papaya bites, and papaya puree) on one occasion each	Not applicable – cross-over trial	Metabolic - **Fasting Blood Glucose**	Blood glucose measurement using HemoCue® cuvette (Helsingborg, Sweden).	**Fasting Blood Glucose** -There were significant differences in the iAUC for the glycemic response over 120 minutes between the treatments. The iAUC for all the fruit types and forms were significantly lower than the glucose beverage (all $p < 0.001$).
Policy											
82	Reid 2007	Non-randomized Uncontrolled before and after study (pre/post test study)	Caribbean / Haiti	Children less than 24 months old - community	International community - would need to investigate beyond this paper to understand who did what.	1593 (not given)	The 'intervention' was the whole of the Haitian population being subject to an international embargo from Oct 1991 to Oct 1994. Few details are given on the embargo, and no details on how it affected the study populations.	'Control' here could be interpreted as the two years before and the two years after the embargo.	Nutrition - **Severe childhood malnutrition**	Weighing children, with 'severe malnutrition' defined as =< -2 Z score of weight for age. Deaths recorded - demographic monitoring system of the Haitian Health Foundation	**Severe childhood malnutrition -** Higher % of severe malnutrition during the embargo period than pre or post embargo. 2. Higher % mortality in the embargo period than pre or post, but not stat sig (at 0.05). Four fold higher risk of death (Cox PH model) in embargo period that in post embargo period

*HbA1C refers to hemoglobin A1c, IG -intervention group, CG – control group, BMI – Body Mass Index, CI – confidence interval, p – probability, IQR -interquartile range, NaFeEDTA - Ferric sodium ethylenediaminetetraacetate, FeFum -ferrous fumarate, CGM - Continuous Glucose Monitoring,
